# Reprogramming Macrophage Metabolism and its Effect on NLRP3 Inflammasome Activation in Sepsis

**DOI:** 10.3389/fmolb.2022.917818

**Published:** 2022-06-29

**Authors:** Ruiheng Luo, Xizhe Li, Dan Wang

**Affiliations:** ^1^ Department of Hematology, The Third Xiangya Hospital, Central South University, Changsha, China; ^2^ Department of Thoracic Surgery, Xiangya Hospital, Central South University, Changsha, China; ^3^ Hunan Engineering Research Center for Pulmonary Nodules Precise Diagnosis & Treatment, Changsha, China; ^4^ National Clinical Research Center for Geriatric Disorders, Changsha, China; ^5^ Department of Dermatology, The Third Xiangya Hospital, Central South University, Changsha, China

**Keywords:** sepsis, NLRP3 inflammasome, metabolism reprogramming, macrophages, targeted therapy

## Abstract

Sepsis, the most common life-threatening multi-organ dysfunction syndrome secondary to infection, lacks specific therapeutic strategy due to the limited understanding of underlying mechanisms. It is currently believed that inflammasomes play critical roles in the development of sepsis, among which NLRP3 inflammasome is involved to most extent. Recent studies have revealed that dramatic reprogramming of macrophage metabolism is commonly occurred in sepsis, and this dysregulation is closely related with the activation of NLRP3 inflammasome. In view of the fact that increasing evidence demonstrates the mechanism of metabolism reprogramming regulating NLRP3 activation in macrophages, the key enzymes and metabolites participated in this regulation should be clearer for better interpreting the relationship of NLRP3 inflammasome and sepsis. In this review, we thus summarized the detail mechanism of the metabolic reprogramming process and its important role in the NLRP3 inflammasome activation of macrophages in sepsis. This mechanism summarization will reveal the applicational potential of metabolic regulatory molecules in the treatment of sepsis.

## Introduction

Sepsis is a complicated syndrome associated with physiological, pathological, and biochemical abnormalities induced by the infection that manifests as life-threatening multiorgan dysfunction (Singer et al., 2016). As a leading public health problem, sepsis is associated with more than $20 billion in total hospital costs in the United States (Torio and Andrews, 2006). The reported incidence of sepsis continues to increase due to the growing number of the aging population and greater awareness (Iwashyna et al., 2012; Gaieski et al., 2013). Although the understanding of the pathological mechanisms has been improved in recent years, targeted treatments are still scant for sepsis (Englert et al., 2019).

The pathogenesis of sepsis is complex, and innate immunity plays a key role. Macrophages are important innate immune cells that significantly affect the occurrence and development of sepsis due to their vital effects on immune homeostasis and the inflammatory response (Gordon and Taylor, 2005; Kumar, 2018; Chen et al., 2021). In sepsis, the metabolic status of macrophages is significantly different from their physiological status, and this abnormal metabolic status regulates their immune function. Pathogen-associated molecular patterns (PAMPs) or damage-associated molecular patterns (DAMPs) and other alarmins can activate macrophages. Activated macrophages suffer from alterations in metabolism and further aggravate or limit the inflammatory response (Chen et al., 2021). Therefore, changes in the metabolic status of macrophages play a complex role in defending against external pressures and stabilizing homeostasis.

Immunometabolism is an interdisciplinary subject combining classical immunology and metabolism and employs the experimental research advances and paradigms of the two fields. Immunometabolism includes two main aspects: cellular immunometabolism studies the changes in immune metabolism that determine the fate of immune cells under different conditions, and tissue immunometabolism studies the effects of immune cells on the metabolism of other tissues and the immune cell-induced regulation of individuals’ adaptation to the environment (Man et al., 2017; Kumar, 2018). Recent studies have shown that immunometabolism plays an important role in infection, tumorigenesis, and many autoimmune diseases (Gaber et al., 2017; Hotamisligil, 2017). Research on immunometabolism in sepsis has also received extensive attention. Metabolism-related enzymes and their intermediates play vital roles in the pathogenesis of sepsis by regulating nucleotide-binding domain, leucine-rich repeat, pyrin domain-containing protein 3 (NLRP3) inflammasome activation and other mechanisms. In this review, we summarized the metabolic reprogramming mechanisms of macrophages in sepsis, with the aim to shed light on novel targets and methods for the treatment of sepsis.

## The Role of Macrophages in Sepsis

Monocytes and macrophages play important roles in host resistance to multiple pathogens and the inflammatory immune responses in sepsis (Lauvau et al., 2015; Kumar, 2018). During the early stage of sepsis, a variety of inflammatory signaling pathways in macrophages are activated, releasing a large number of inflammatory mediators and aggravating the progression of sepsis. During this process, M1 macrophages are dominant (Zubair et al., 2021). The late stage of sepsis, often accompanied by immunosuppression or paralysis, is mainly caused by the apoptosis of conventional T cells and the upregulation of regulatory T cells (Tregs), in which macrophages also play an important role (Zubair et al., 2021). This endotoxin tolerance exhibited by macrophages is the result of a phenotypic shift M1 to M2 macrophages or alternatively activated macrophages (AAMs), which contributes to the immunosuppression observed during the later stage of sepsis (Wang and Deng, 2008; Delano and Ward, 2016).

Studies have shown that in different stages of sepsis, especially in the early stage, the regulatory function of the metabolic pathway of macrophages is significantly changed (Artyomov et al., 2016; Arts et al., 2017). Macrophage glycolysis, which includes the pentose phosphate pathway (PPP), and the tricarboxylic acid (TCA) cycle are both altered during sepsis. Moreover, changes in cellular glucose metabolism pathways, such as in the key enzymes and intermediates, could impact the progression of sepsis by regulating NLRP3 inflammasome activation, multi-inflammatory factor release, and others (Kumar, 2018).

## Macrophage Metabolic Reprogramming and Adaptation in Sepsis

Glucose metabolism is the core metabolic pathway that is mainly achieved by glycolysis, the PPP, and the TCA cycle. The following is a summary of changes in macrophage glycolysis and the TCA cycle in sepsis.

### Glycolysis

Glycolysis is a key step in cellular glucose metabolism, enabling cells to obtain energy in the form of ATP. Glucose in the serum is transferred to cells by glucose transporters (GLUTs) and converted to pyruvate through a series of enzymatic reactions during anaerobic glycolysis (Freemerman et al., 2014). The resulting pyruvate can be converted to lactate by the anaerobic glycolysis pathway to produce 2 ATPs or converted to acetyl-CoA to enter the TCA cycle and ultimately produce 36 ATPs through oxidative phosphorylation (OxPhos). Although ATP production is consumed during glycolysis, it is could be quickly restored via glycolysis to fulfil the short-term energy. In contrast, the TCA cycle and OxPhos produce ATP at a relatively slow rate despite high production. The Warburg effect refers to the transformation of cell metabolism from OxPhos to glycolysis to produce ATP at a faster speed for cell energy requirements. This phenomenon was initially observed in tumor cells and has subsequently been demonstrated in activated immune cells, including macrophages (Hsu and Sabatini, 2008).

During sepsis, glycolysis in highly activated macrophages is significantly upregulated, and the molecular mechanisms are currently well-documented. Studies have shown that hypoxia-inducible factor (HIF-1α) plays a key role in promoting glycolysis, and the up-regulation of the Akt-mTOR signaling pathway targets HIF-1α to promote glycolysis in activated macrophages (Cheng et al., 2014). In addition, lipopolysaccharide (LPS), a key pathogenic factor of sepsis, could promote the expression of GLUT1 and GLUT3 and thus increase the glucose uptake of macrophages (Fukuzumi et al., 1996; Reddy et al., 2010). In addition, LPS has also been reported the expression of key enzymes in the glycolysis pathway, such as hexokinase (HK) and fructose 2,6-bisphosphate (F2,6BP) (Tavakoli et al., 2013). Likewise, zinc fingers and homeoboxes (Zhx2) have been confirmed to be involved in sepsis by promoting the expression of 6-phosphofructo-2-kinase/fructose-2,6-biphosphatase 3 (Pfkfb3), thereby increasing glycolysis (Wang et al., 2020). Lactate, the end product of glycolysis, is significantly accumulated in macrophages after LPS treatment (Yang et al., 2014). The increase of lactate caused by macrophage glycolysis is thought to partly contribute to the accumulation of lactate in tissues during sepsis. As a result, lactate is known as an independent risk factor for sepsis-inducing death (James et al., 1996; Gomez and Kellum, 2015). In addition, caspase-1 has been proven to be a key actuator of inflammation, pyroptosis, and sepsis. Studies have shown that caspase-1 can cleave several key enzymes in glycolysis, including aldolase, triose-phosphate isomerase, glyceraldehyde-3-phosphate dehydrogenase, alpha-enolase, and pyruvate kinase, which are further involved in the occurrence of sepsis and septic shock (Shao et al., 2007).

Based on the important role of macrophage glycolysis in the occurrence of sepsis as summarized above, the targeted regulation of glycolysis can impact the progression of the disease. Studies have shown that exosomes released from bone marrow mesenchymal stem cells (BMSCs) can reduce macrophage glycolysis by inhibiting HIF-1α, thereby attenuating sepsis-inducing lung injury (Deng et al., 2020). Likewise, glycolytic inhibitor 3PO could robustly improve the prognosis of acute lung injury (ALI)-induced by sepsis via reducing the lung inflammation and histopathological changes (Gong et al., 2017). 2-deoxy-D-glucose (2-DG), an inhibitor of aerobic glycolysis, has been found to alleviate sepsis-induced kidney injury by regulating autophagy (Tan et al., 2021). The molecular mechanism is related to the upregulated sirtuin 3 (SIRT3) and phosphorylation-AMP-activated protein kinase (p-AMPK). Xijiao Dihuang decoction (XJDHT) is a traditional Chinese medicine (TCM) formula. XJDHT is proven to restrain the activation of TLR4-HIF-1α signal pathway, reduce aerobic glycolysis and subsequently prompt the survival rate of sepsis (Lu et al., 2020).

In addition, PPP is also a vital metabolic pathway derived from glycolysis to achieve glucose oxygenolysis. PPP is divided into an oxidized branch and a non-oxidized branch, which can generate NADPH for fatty acid synthesis and ribose-5-phosphate for nucleoside synthesis. Moreover, the non-oxidized branch can transport metabolites back to glycolysis. Studies have shown that G6PD, a key enzyme in the PPP, was significantly upregulated in macrophages from a mouse sepsis model induced by intraperitoneal LPS injection (Arts et al., 2017).

### Lipid Metabolism

Lipids included fatty acids, phospholipids, cholesterol and other different types of molecules (Meyers and Zhu, 2020). Besides of serving as important sources of energy when nutrients are limited or as a key component of different membranes, lipids, also are regarded as key regulators of macrophage function, especially in the presence of abnormal activation of inflammatory pathways, such as sepsis (Meyers and Zhu, 2020). The results of a metabolomics and proteomics study on 1000 sepsis patients’ plasma showed that, nine fatty acid transport proteins were reduced in non-survivors, indicated that fatty acid *ß*-oxidation is severely impaired in sepsis non-survivors (Langley et al., 2013). Thus, fatty acid metabolism could be one of the most promising metabolic biomarkers for predicting survival rate for sepsis patients (Langley et al., 2013). In rat sepsis model, several fatty acids were shown to be significantly increased in serum 2 h after cecal ligation (Wei et al., 2016).

Besides as a biomarker, the fatty acid has been explored as a potential treatment in sepsis. In two classic models of sepsis, murine cecal ligation (CLP) model and intraperitoneal injection with *E. coli*, adding exogenous stearoyl-GPC has been shown to decline the death rate in septic mice (Yan et al., 2004). Supplementation with ω-3-fatty acid supplements has been shown fewer secondary infections and shorter ICU time in sepsis patients than the patients without extra supplements (Galbán et al., 2000; Beale et al., 2008; Al-Biltagi et al., 2017).

### Amino Acid Metabolism

Disorder of amino acid metabolism is also an important feature of sepsis. Different types of amino acids vary to different degrees in severe sepsis patients. the levels of 3-methyl-L-histidine, *a*-aminoadipic acid, *a*-amino-n-butyric acid, argininosuccinic acid, *ß*-aminoisobutyric acid, carnosine, cystathionine, glutamine, phenylalanine, and proline increased, while the levels of arginine, asparagine, aspartic acid, cystine, glutamic acid, leucine, serine, taurine, and tryptophan decreased in sepsis patients (Freund et al., 1979). A new study has found that level of branched-chain amino acids (BCAA) was significantly lower in sepsis non-survivors compared with survivors and may be a promising early biomarker for predicting outcome in sepsis patients (Reisinger et al., 2021). The prevailing interpretation of imbalance of amino acid levels in sepsis and the underlying mechanisms remain to be further investigated (Su et al., 2015; Reisinger et al., 2021).

### TCA Cycle/OxPhos

The TCA cycle is an important part of glucose metabolism that connects different metabolic pathways. Pyruvate, the product of glycolysis, is the raw material of the TCA cycle. Pyruvate dehydrogenase catalyzes the conversion of pyruvate to acetyl-CoA, which subsequently enters the TCA cycle. The TCA cycle can produce a large amount of ATP to sustain normal body functions, and it is also a common pathway in glucose metabolism, lipid metabolism and amino acid metabolism. The metabolites of the TCA cycle can participate in the synthesis of various fats and amino acids.

As previously mentioned, LPS-activated macrophages change glucose metabolism, manifested by increased glycolysis and decreased TCA cycle/OxPhos. Although the TCA cycle was reduced, LPS-stimulated macrophages were found to be significantly enriched in various TCA cycle metabolites, including succinate, malate, fumarate, and others (Tannahill et al., 2013). Citrate and oxaloacetic acid were proven to be increased in the experimental rat model of *Klebsiella* pneumonia bacteremia (Dong et al., 2012). In addition, recent studies showed that itaconate, another intermediate of the TCA cycle, is significantly increased in LPS-activated macrophages (Bambouskova et al., 2018; Mills et al., 2018).

## Key Enzymes and Metabolic Intermediates Emerging as Vital Players in Macrophage Functions in Sepsis

It has been summarized above that the metabolism of macrophages is changed in sepsis, and the altered metabolism will further affect their immune effect through key enzymes or intermediate metabolites. In the following section, we will summarize how the molecular mechanisms of the key enzymes and intermediate metabolites of glucose metabolism affect the immune effect of macrophages.

### The Critical Processes of NLRP3 Inflammasome Activation

Inflammasomes are a class of pattern-recognition receptors (PPRs) located in the cytoplasm and were first detected in 2002 (Martinon et al., 2002). Inflammasomes are multi-protein complexes that can be classified as NLRP1, NLRP3, NLRC4, or AIM2 based on the different kinds of nucleotide-binding oligomerization domain (NOD)-like receptors (NLRs) (Xu et al., 2019). The NLRP3 inflammasome is composed of NLRP3 (sensor), ASC (adaptor), and caspase-1 (effector), which is the most intensely studied inflammasome currently. A previous study summarized the molecular mechanism of NLRP3 inflammasome activation and its research progress in inflammation-related diseases (Martinon et al., 2002).

It is believed that the progress of activation of the NLRP3 inflammasome requires two signals. The priming signal is composed of the upregulation of NLRP3 and proinflammatory cytokines such as IL-1β. The process is induced by the activation of NF-kB in the presence of microbial components or endogenous molecules. The robust activation of these molecules can elevate the expression of NLRP3. Recent studies have shown that the NLRP3 inflammasome is activated by a variety of stimuli, including PAMPs (LPS, nigericin, gramicidin, etc.) or DAMPs (ATP, cholesterol crystals, monosodium urate crystals, alum, silica, etc.), and many kinds of posttranscriptional modifications (PTMs) participate in the priming step. Among them, phosphorylation and ubiquitination were the most common (Fernandes-Alnemri et al., 2007; Lamkanfi, 2011).

The second signal, also known as the activating step, is currently obscured because of the diversity of NLRP3 activation stimuli. Many common cellular signals can trigger NLRP3 activation (Rathinam et al., 2012). Changes in intracellular and extracellular ion content, such as K^+^ or Ca^2+^, or mitochondrial dysfunction and lysosomal rupture, are all thought to be critical triggers of NLRP3 activation.

The activated NLRP3 inflammasome cleaved caspase-1 and promotes interleukin (IL)-1β maturation. Meanwhile, gasdermin D (GSDMD) is cleaved, which subsequently causes pore formation in the cell membrane and mediates cell pyroptosis. In addition to the canonical pathways, caspase-11 can also induce NLRP3 activation. Once LPS is delivered into the cytosol, Caspase-11, senses the cytosolic LPS and cleaves the substrate GSDMD and using the efflux of K^+^ to complete the activation of NLRP3 by inducing the opening of the pannexin-1 channel (Kayagaki et al., 2011). Recent studies revealed that these standard NLRP3 activation signals were also regulated by metabolic intermediates and enzymes, which we will discuss in the following section.

### Key Enzymes and Metabolic Intermediates Regulating NLRP3 Inflammasome Activation

As mentioned above, the activated NLRP3 inflammasome mediates the release of inflammatory factors and the occurrence of pyroptosis, and therefore is thought to play an important role in the development and progression of sepsis.

Studies have shown that NLRP3 inflammasome expression and activation are significantly enhanced in the peripheral blood monocytes of septic patients (Esquerdo et al., 2017; Martínez-García et al., 2019). The knockout of NLRP3 or inhibition of its activation significantly reduces the release of inflammatory mediators, decreases multiple organ failure (MOF) in sepsis, and improves the survival rate of sepsis (Sutterwala et al., 2006; Mao et al., 2013; Danielski et al., 2020). Conversely, upregulation of NLRP3 inflammasome activation can aggravate MOF in sepsis and increase sepsis mortality (Shi et al., 2020). Likewise, our previous study also confirmed that regulation of NLRP3 inflammasome activation can affect the occurrence and development of sepsis (Yu et al., 2019a; Wang et al., 2021). Therefore, the NLRP3 inflammasome plays a key role in sepsis, and regulation of its activation can affect the progression of sepsis. Recently, an increasing number of studies have found that the key enzymes and metabolites of glucose metabolism in macrophages are involved in the pathogenesis of sepsis by regulating the activation of the NLRP3 inflammasome.

Studies have shown that HK1, an HK isozyme, upregulates NLRP3 inflammasome activation to augment the secretion of IL-1β and IL-18 in macrophages (Moon et al., 2015a). Based on previous studies showing that LPS treatment significantly promoted glycolysis in macrophages, researchers found that inhibition of glycolysis could reduce the production of the inflammatory factor IL-1β(Moon et al., 2015a). Jong-Seok Moon *et al* also found that the molecular mechanism of glycolysis impacting IL-1β production is attributed to the upregulation of NLRP3 inflammasome activation controlled by the mTORC1-dependent glycolysis metabolic pathway. mTORC1 promotes NLRP3 activation by promoting the expression of HK1, a key enzyme in the glycolysis pathway that converts glucose to glucose-6-phosphate, while suppression of HK1-dependent glycolysis restrains NLRP3 inflammasome activation (Moon et al., 2015a).

The M2 isoform of the pyruvate kinase muscle isozyme (PKM2), is a key rate-limiting enzyme in the glycolysis pathway, catalyzing the conversion of phosphoenolpyruvate and ADP to pyruvate and ATP. Studies have shown that the expression of PKM2 is significantly increased in LPS-stimulated macrophages (Palsson-McDermott et al., 2015a). Inhibiting PKM2 activation reduced IL-1β release and improved survival in septic mice while promoting PKM2 activation increased IL-1β levels and significantly reduced the host immune response in a sepsis model (Yang et al., 2014; Palsson-McDermott et al., 2015a). Subsequent studies have shown that PKM2 promotes IL-1β, IL-18, and HMGB1 release by upregulating the activation of the NLRP3 inflammasome (Xie et al., 2016). The molecular mechanism is related to the promotion of phosphorylation by PKM2. EIF2AK2 is closely related to NLRP3 inflammasome activation (Lu et al., 2012). *In vivo*, inhibition of PKM2-EIF2AK2 signaling protects mice from lethal endotoxemia and polymicrobial sepsis (Xie et al., 2016).

Lactate, a product of glycolysis, was shown to promote IL-1β and HMGB1 release in LPS-stimulated macrophages (Yang et al., 2014). Further studies revealed that the molecular mechanism was via the promotion of the activation of the NLRP3 inflammasome by lactate (Xie et al., 2016). Caspase-1 deficiency reduced the lactate-induced IL-1β release in macrophages under LPS stimulation, while both caspase-1 and caspase-11 deficiency completely blocked the IL-1β release induced by lactate after LPS treatment (Xie et al., 2016).

In addition, citrate is a metabolic intermediate of the TCA cycle and plays a critical role in cellular energy metabolism as a substrate. As mentioned above, citrate is enriched in LPS-treated macrophages. It has been recently found that enriched citrate can promote subsequent inflammatory responses (Duan et al., 2021). Citrate has been shown to increase the expression of NLRP3 and pro-IL-1β and subsequently augment NLRP3 inflammasome activation. Abnormal NLRP3 inflammasome activation was considered to then aggravate lung injury induced by LPS(Duan et al., 2021).

However, itaconate, a TCA cycle-related product, was found to reduce IL-1β and IL-18 release by inhibiting NLPR3 activation (Lampropoulou et al., 2016). Fumarate, an intermediate of the TCA cycle, accumulated in LPS-stimulated macrophages. Studies have shown that fumarate inhibits both canonical and noncanonical pathway activation by blocking the interaction of GSDMD with caspases, limiting the capacity to induce cell death (Humphries et al., 2020).

As indicated above, abnormally increased glycolysis-related enzymes and intermediate products of TCA cycle metabolism affect the inflammatory process of macrophages by regulating the activation of the NLRP3 inflammasome. Targeting the key signaling pathways is expected to lead to advances in sepsis treatment.

Besides glycolysis metabolism, key enzymes in lipid metabolism also play an invaluable role in regulating NLRP3 inflammasome activation. UCP2, which appears mainly in innate immune cells, has higher expression in sepsis patients compared with non-sepsis patients (Fleury et al., 1997; Moon et al., 2015b). In UCP2-deficient macrophages, lipid synthesis was suppressed due to the downregulation of fatty acid synthase (FASN) which is a key regulator of fatty acid synthesis (Moon et al., 2015b). UCP2-deficient or FASN inhibition suppressed NLRP3 inflammasomes activation by decreasing NLRP3 and pro-IL-1β gene expression in macrophages and has a protective effect on the endotoxin shock model and the CLP model (Moon et al., 2015b).

### Other Mechanisms of Key Enzymes and Metabolic Intermediates Impacting Macrophage Functions in Sepsis

In addition to the NLRP3 inflammasome, key enzymes and metabolic intermediates of cellular glucose metabolism also affect macrophage function through a variety of molecular mechanisms, thus contributing to the occurrence and development of sepsis.

PKM2 not only regulates macrophage function by promoting NLRP3 inflammasome activation but also promotes macrophage activation through other signaling pathways. PKM2 was shown to interact with HIF-1α, and HIF-1α subsequently combined with the IL-1β promoter to induce IL-1β expression (Yang et al., 2014). PKM2 induces various proinflammatory factors and promotes the anti-inflammatory state of macrophages, also known as the M1 macrophage phenotype (Palsson-McDermott et al., 2015b).

Succinate, an intermediate of the TCA cycle, plays a key role in regulating macrophage activation. Studies have shown that succinate accumulation induced by LPS in macrophages contributes to the production of IL-1β. The molecular mechanism is that succinate stabilizes HIF-1α and further upregulates IL-1β transcription (Tannahill et al., 2013). In addition, Evanna L Mills et al. found that in LPS-stimulated macrophages, succinate dehydrogenase (SDH) induced the mitochondrial oxidation of succinate, which led to the production of mitochondrial reactive oxygen species (mtROS), an important proinflammatory mediator (Mills et al., 2016). Inhibition of succinate oxidation protected mice from endotoxin-induced lethalitye (Mills et al., 2016). However, succinate was shown to play a potential role in limiting inflammation. Succinate receptor 1 (SUNCR1) is an extracellular sensor of succinate. The myeloid-specific SUNCR1 knockout induced the expression of various pro-inflammatory factors, including IL-1β, while activated SUNCR1 led to an anti-inflammatory state and caused macrophage polarization (Keiran et al., 2019).

In addition, itaconate inhibits macrophage inflammation through a variety of molecular mechanisms, although it does not regulate NLRP3 inflammasome activation. Itaconate decreased the alkylation of Keap1 cysteine residues, which was related to the translocation and activation of Nrf2. Moreover, itaconate blocked the ATF3/eIF2a signaling pathway to restrain the production of proinflammatory mediators. In addition, accumulated IFN-β was shown to increase the accumulation of itaconate, while itaconate was shown to inhibit the expression of IFN-β to control the inflammatory response (Yu et al., 2019b).

### Potential Anti-Sepsis Drugs Targeting Glucose Metabolic

It has been concluded that the glucose metabolism of macrophages is disturbed in sepsis, and glucose metabolism further influences the occurrence and development of sepsis by regulating NLRP3 inflammasome activation. Therefore, in recent years, more and more studies have found that a variety of drugs can play a therapeutic role in sepsis by interfering with glucose metabolism.

### 2‐deoxyglucose (2‐DG)

2-DG is a nonmetabolizable glucose analog that inhibits glycolysis by acting on a rate-limiting enzyme, hexokinase. The mechanism is that 2-DG is phosphorylated by hexokinase to 2-DG-P, which cannot be further metabolized to produce ATP. Based on the important role of glycolysis in sepsis, whether 2-DG can affect the development of sepsis is gradually being revealed. Studies showed that 2-DG treatment reduced pro-inflammatory factors expression, inflammatory cell accumulation, lung tissue pathological injury, and lethality in sepsis *in vivo* (Zhong et al., 2019). Further exploration found that 2-DG inhibited NLRP3 inflammasome activation in macrophages during sepsis (Zhong et al., 2019). Besides, 2-DG was proved to improve the survival of sepsis as well as reduce sepsis-related kidney, liver, and cardiac injury by suppressing IL-1β and lactate levels (Zheng et al., 2017). *Chuyi* Tan et al. found that sepsis-induced acute kidney injury could be alleviated by 2-DG via the lactate/SIRT3/AMPK pathway (Tan et al., 2021). To sum up, 2-DG plays an important role in sepsis and sepsis-related multi-organ dysfunction, which suggested that 2-DG has the potential to be a treatment for sepsis.

### Terazosin

Terazosin, an alpha1-adrenergic receptor agonist, is mainly used for the treatment of benign prostatic hyperplasia in the elderly. Recently studies showed that terazosin increased sepsis survival rate and decreased organ injury *in vivo*. The molecular mechanism was that terazosin acted on the key glycolysis enzyme PGK1, which was a novel target (Chen et al., 2015). This study suggests that terazosin may be useful in the treatment of sepsis.

### Insulin

Insulin is the only physiological glucose-lowering hormone and is often used in the treatment of diabetic patients. However, some research has revealed that insulin has anti-inflammatory effects. Pretreatment with insulin inhibited inflammatory response by suppressing the activation of NF-κB and expression of pro-inflammatory cytokines (Chen et al., 2014; Sun et al., 2014; Huang et al., 2020). A recent study also found that insulin inhibited NLRP3 inflammasomes activation by reducing the oligomerization of the ASC(Chang et al., 2020). Thus, insulin is a promising drug for sepsis.

### Metformin

Metformin, as the first-line medication for the treatment of diabetes, inhibited NLRP3 inflammasome activation and IL-1β production in cultured and alveolar macrophages, thus attenuating LPS and SARS-CoV-2-induced acute respiratory distress syndrome (ARDS) (Xian et al., 2021). The result of phase II randomized controlled trial suggested that metformin dose not reduce the morbidity of severely burned patients who suffered sepsis, indicated further study is needed. Besides several infectious diseases, metformin has also been discovered to prevent NLRP3 inflammasome activity both in periodontitis (Li et al., 2016) and psoriasis (Tsuji et al., 2020).

## Conclusion and Future Directions

**FIGURE 1:**
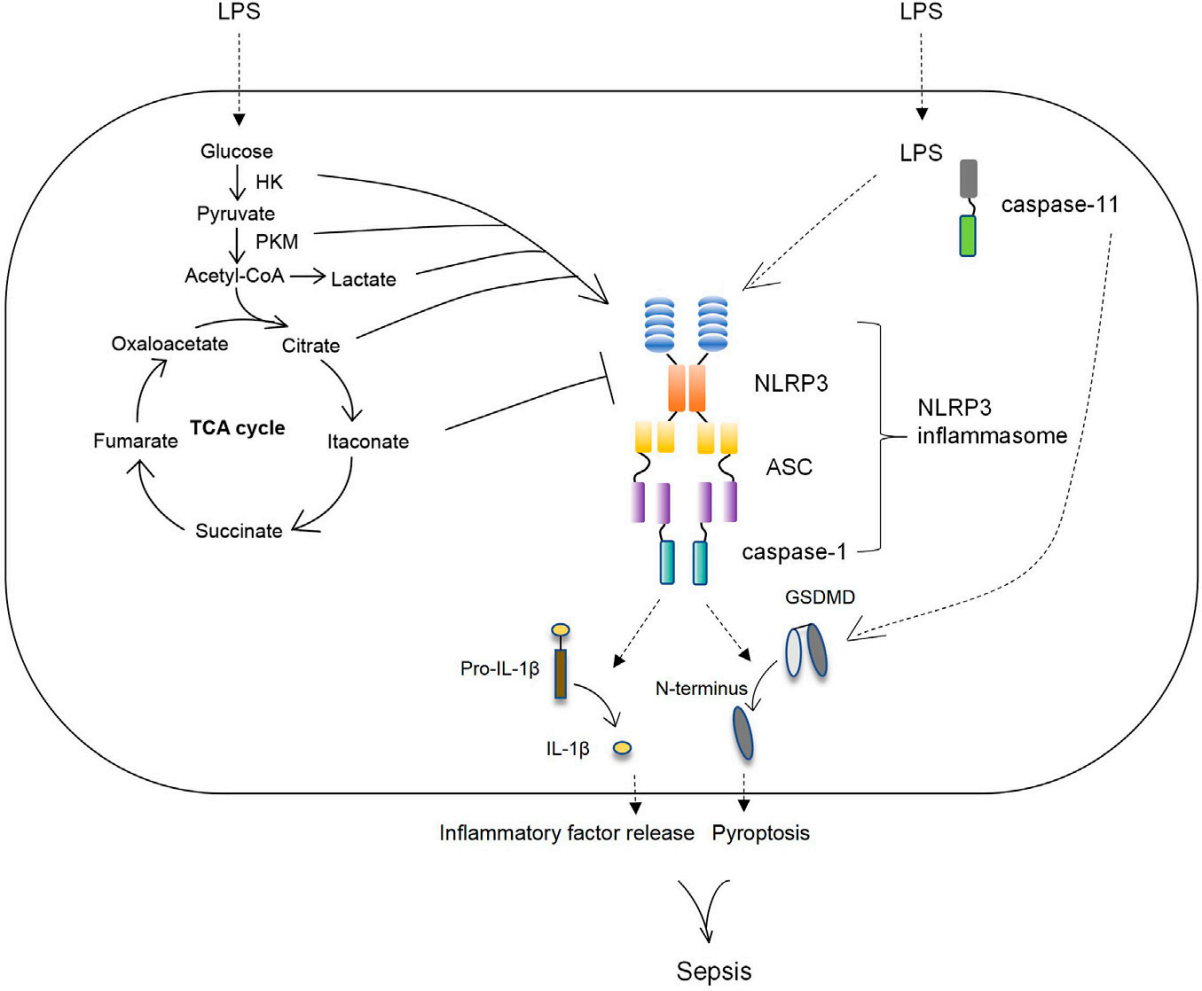
The key enzymes and metabolic intermediates participated in the activation of NLRP3 inflammasome. During sepsis the glucose metabolism of macrophage was changed, manifested by increased glycolysis and decreased TCA cycle/OxPhos as well as enriched various TCA cycle metabolites. Subsequently, the key enzymes and intermediates of glucose metabolism in macrophage regulated the activation of NLRP3 inflammasome to affect macrophage functions. HK and PKM, the key enzymes of glycolysis, positively upregulated NLRP3 inflammasome. Lactate, the metabolism of glycolysis, as well as citrate, the metabolism of TCA cycle, increased NLRP3 inflammasome activation. Itaconate, the intermediate of TCA cycle, downregulated NLRP3 inflammasome activation.

This review summarizes changes in the immune metabolism of macrophages, which play a key role in sepsis (Figure 1). In this paper, two aspects are discussed: first, the changes of macrophage metabolism including glycolysis and TCA cycle in sepsis are summarized; next, we review the key enzymes and metabolites of altered cell metabolism that affect macrophage immune status by regulating NLRP3 inflammasome activation and other mechanisms; besides, the potential drugs targeted the immune metabolism to treat sepsis are summarized. This summary of the research progress on the immune metabolism of macrophages in sepsis is helpful to deeply understand the pathogenesis of sepsis and provide targets and a theoretical basis for the treatment of sepsis. However, the molecular mechanism of a large number of cellular metabolic enzymes and intermediates regulating macrophage immune status is still unclear. Further exploration of new molecular mechanisms is expected to allow for more specific and precise interventions to influence the immune response in macrophage metabolic disorders, improve the effectiveness of the interventions, and reduce side effects.
